# Retraction Note: The novel circular RNA circ-CAMK2A enhances lung adenocarcinoma metastasis by regulating the miR-615-5p/fibronectin 1 pathway

**DOI:** 10.1186/s11658-024-00604-7

**Published:** 2024-06-11

**Authors:** Jiahui Du, Guangzhao Zhang, Hongli Qiu, Haifeng Yu, Wuying Yuan

**Affiliations:** Minimally Invasive Surgery, Henan Provincial Chest Hospital, No. 1 Weiwu Road, Jinshui District, Zhengzhou, 450000 People’s Republic of China

**Retraction Note: Cellular & Molecular Biology Letters (2019) 24:72** 10.1186/s11658-019-0198-1

The Editor-in-Chief has retracted this article after concerns were raised about the data reported. The journal was initially contacted by the authors to report concerns about the reproducibility of their results. The authors were then unable to provide raw data upon request by the publisher. The Editor-in-Chief no longer has confidence in the reliability of the findings and conclusions of this article.

All authors agree with this retraction.

